# When “Bouba” equals “Kiki”: Cultural commonalities and cultural differences in sound-shape correspondences

**DOI:** 10.1038/srep26681

**Published:** 2016-05-27

**Authors:** Yi-Chuan Chen, Pi-Chun Huang, Andy Woods, Charles Spence

**Affiliations:** 1 Department of Experimental Psychology, Oxford University, Oxford, UK; 2 Department of Psychology, National Cheng Kung University, Tainan, Taiwan; 3 Xperiment, Surrey, UK

## Abstract

It has been suggested that the Bouba/Kiki effect, in which meaningless speech sounds are systematically mapped onto rounded or angular shapes, reflects a universal crossmodal correspondence between audition and vision. Here, radial frequency (RF) patterns were adapted in order to compare the Bouba/Kiki effect in Eastern and Western participants demonstrating different perceptual styles. Three attributes of the RF patterns were manipulated: The frequency, amplitude, and spikiness of the sinusoidal modulations along the circumference of a circle. By testing participants in the US and Taiwan, both cultural commonalities and differences in sound-shape correspondence were revealed. RF patterns were more likely to be matched with “Kiki” than with “Bouba” when the frequency, amplitude, and spikiness increased. The responses from both groups of participants had a similar weighting on frequency; nevertheless, the North Americans had a higher weighting on amplitude, but a lower weighting on spikiness, than their Taiwanese counterparts. These novel results regarding cultural differences suggest that the Bouba/Kiki effect is partly tuned by differing perceptual experience. In addition, using the RF patterns in the Bouba/Kiki effect provides a “mid-level” linkage between visual and auditory processing, and a future understanding of sound-shape correspondences based on the mechanism of visual pattern processing.

Constantly bombarded by massive amounts of sensory information, the human brain tries to make sense of the world by associating the signals in different sensory modalities that likely belong to the same objects and events. Crossmodal correspondences, such as found between larger (smaller) objects and lower- (higher-) pitched sounds^1^, provide an important constraint that may help observers to correctly associate the appropriate unisensory signals, thus helping to solve the crossmodal binding problem. Intriguingly, though, the evidence suggests that we remain mostly unaware of the existence of crossmodal correspondences (see Spence, for a review^2^). One of the most well-established crossmodal correspondences between sounds and shapes is that the majority of people match the nonsense word “Bouba” with rounded patterns while matching the nonsense word “Kiki” with more angular patterns instead (see Fig. 1 for examples).

The Bouba/Kiki effect, first demonstrated almost 90 years ago^3^,^4^,^5^, has since been repeatedly been verified in various groups of participants, including infants and young children^6^,^7^,^8^,^9^, as well as in various populations that are remote from Western culture^10^,^11^,^12^. The evidence therefore suggests that this particular sound-shape correspondence is *universal*^13^,^14^. To date, however, sound-shape correspondences have primarily been demonstrated using arbitrary visual patterns and some of their variations. Hence, little is known concerning the specific visual characteristics that may underlie this particular correspondence; in turn, the role of visual pattern perception in the Bouba/Kiki effect is currently unclear.

Researchers have suggested that the crossmodal correspondence between the speech sounds of Bouba-Kiki and rounded-angular shapes may be a type of natural mapping. According to the dominant view, the effect may reflect a natural constraint embedded in language, known as *sound symbolism*^15^,^16^. That is, for example, in different languages, such as English words (e.g., *round* and *spiky*) and the Chinese characters (e.g., 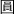 [yuan2]: *round*, and 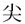
[jian1]: *spiky*; the number in the square brackets demotes the tone of the pronunciation in Mandarin) that are used to describe the rounded and angular shapes happen to consist of the vowels /u/ and /i/, respectively, and the vowels (as compared to consonants) are the more influential phonemes in the Bouba/Kiki effect^8^,^17^,^18^. Namely, visual shapes (rounded or angular) seem to be associated with lip movements when uttering the vowel /u/ or /i/ (rounded or stretched lips). One plausible neural mechanism that may underlie the phenomenon of sound symbolism involves the sensory-motor connections that exist between cortical visual areas and motor areas. An alternative suggestion is that they might also be mediated by mirror neurons that connect the observation of others’ lip shapes and the observer’s own motor representations^8^,^19^.

Others have suggested that the Bouba/Kiki effect may be associated with statistical learning processes to form a type of metaphorical representations in human perception^20^,^21^,^22^, with rounded shapes associated with lower-pitched sound whilst angular shapes are associated with higher-pitched sound instead^23^,^24^,^25^. Indeed, the sound “Kiki” consists of stronger auditory signals at high frequency band above 10,000 Hz as compared to “Bouba” (see the spectrograms of “Bouba” and “Kiki” used in the present study in Fig. 2). Hence, the fact that angular shapes are associated with “Kiki” can be partly attributed to the acoustic features of the latter being composed of high-pitch sound. It is thought that such crossmodal correspondences between visual and auditory features are established on the basis of their statistical co-occurrence in daily perceptual experience^2^; in addition, certain abstract (or modality-general) semantic/conceptual representations associating various attributes of an object may be formed (such as that sharp objects like knives produce high-pitched sound^26^).

The above two accounts for the Bouba/Kiki effect should not necessarily be thought of as being mutually exclusive, and hence their influences may be hard to distinguish – both can be used to explain the Bouba/Kiki effect that has, as we have seen, been suggested to be universal^10^,^19^. Nevertheless, the metaphorical perception account has a perceptual basis in terms of how a visual pattern is processed or perceived. That is, whether a visual pattern would be associated with “Bouba” or “Kiki” should be determined by how its features are processed by an observer. To examine this hypothesis, two novel approaches are adopted in the current study: First, a series of patterns was generated using the formula of radial frequency (RF) patterns^27^. In this case, we systematically manipulated the features of visual shapes in order to examine the Bouba/Kiki effect. Second, the Bouba/Kiki effect was compared in Eastern and Western participants who, it has been demonstrated previously, exhibit different perceptual styles; specifically, Easterners show a tendency to process visual patterns or scenes holistically, whereas Westerners, process them more analytically^28^,^29^.

## Radial frequency (RF) patterns. 

RF patterns are closed-contours with sinusoidal modulations along the circumference of a circle^27^ (see examples in Fig. 3). RF patterns are considered to be an example of “mid-level representation” in the hierarchy of visual feedforward processing and have been widely used in studies of shape perception. It has been suggested that the representation of RF patterns is formed by combining the local filter responses in the early visual area (V1) where the visual patterns are decoded into various orientation and spatial frequencies^30^. The pooling of such information plausibly occurs at V4 where neurons have larger receptive fields that are tuned to radial and concentric patterns^31^,^32^,^33^.

RF patterns therefore provide a novel and useful tool to study crossmodal sound-shape correspondence and offer the advantage that the features of RF patterns can be manipulated systematically by changing corresponding parameters in the mathematical function. Hence, we can create several patterns with step-by-step changes along the predesignated dimensions and then test how participants’ matching shifts from “Bouba” to “Kiki”.

## Cultural differences in perception. 

Human perception, perhaps surprisingly, has been demonstrated to be affected by cultural background. For example, by comparing two of the world’s distinct cultures, Easterners are suggested to be collectivist and to pursue group harmony. They tend to associate visual objects across a broader region of the visual field, or to attend to their relationships, during perceptual processing^29^,^34^,^35^; Westerners, by contrast, are thought to be individualistic and to emphasize personal agency. Thus they tend to focus on the foreground object that is somehow detached from the context^29^,^35^. In a test using Navon figures (e.g., a holistic, large letter E composed of small elements - letter H’s), for example, Easterners demonstrate an advantage when responding to the holistic letter E in terms of both response time and accuracy measures as compared to Westerners^28^. On the basis of such evidence, it has been argued that Easterners’ and Westerners’ perceptual processing styles can be characterized as *holistic* and *analytic*, respectively^29^,^36^.

To summarize, this is the first study of its kind to use RF patterns to systematically manipulate the features of visual patterns in order to examine the Bouba/Kiki effect in different cultures. Easterners and Westerners, who tend to notices the holistic features or individual elements of a visual pattern, respectively, may demonstrate certain differences in matching a given RF pattern to Bouba or Kiki. We conducted the on-line study in order to recruit a large number of participants in Western and Eastern culture^37^.

## Methods

### Participants

Two groups of participants took part in the study: 150 participants (age range: 19–68 years) recruited from Amazon’s Mechanical Turk (US group). They received an on-line shopping voucher in return for their participation. The other group consisted of 88 undergraduate students (age range: 18–22 years) from National Cheng Kung University in Taiwan who received additional course credit in return for their participation (Taiwanese group). Five additional participants in the US group and one in the Taiwanese group failed to complete the experiment and so their data were excluded from further analysis. All of the participants were naïve as to the purpose of the study. The participants gave their informed consent before the experiment. All of the procedures were carried out in accordance with the Declaration of Helsinki and were approved by the ethical committee in Medical Sciences Inter Divisional Research Ethics Committee, University of Oxford (MSD-IDREC-C1-2014-141), and in the Department of Psychology, National Cheng Kung University.

### Stimuli and Design

Three RF pattern dimensions were manipulated (see Fig. 3): Frequency (the number of sinusoidal modulations per circle), Amplitude (the magnitude of the sinusoidal modulations deviating from a circle; from 0 to 1), and Spikiness were manipulated by increasing the number of harmonics of triangular wave forms added on top of each sinusoidal modulation. The equation to plot RF patterns can be defined as a function of polar angle (θ):


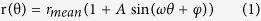


where *r*_*mean*_ is the radius of the base circle, *A* is the amplitude of the sinusoidal modulation, *ω* is the frequency, and *φ* is the phase of the sinusoids. The harmonics of a triangular wave form was added using the following equation:


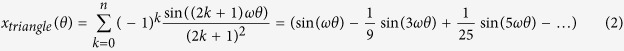


Thus the stimulus used can be simplified as follows:


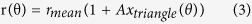


There were six level of Frequency (4, 5, 6, 7, 8, and 9), four levels of Amplitude (0.1, 0.2, 0.3, and 0.4), and three levels of Spikiness (0, 1, and 30). These levels were chosen based on the basis of pilot results (see the Pilot Experiment 1 in the Supplementary Materials). Furthermore, our pilot study also demonstrated that the tendency of matching RF patterns to “Bouba” or “Kiki” did *not* vary with the size and the left-right symmetry of the RF patterns, providing a contrast showing that Frequency, Amplitude, and Spikiness are truly essential factors in the current study (see Pilot Experiment 2 in the Supplementary Materials). Each RF pattern consisted of a black outline presented against a white background.

The auditory stimuli consisted of the spoken nonsense words “Bouba” and “Kiki” as recorded by a female native English speaker (32 bit mono; 44,100 Hz digitization). Each non-word was recorded three times with slightly different speeds and tones (see Fig. 2). All six sound files were edited to the same length (400 ms) and their sound pressure level (in terms of the value of root mean square) were equalized. The experiment was conducted on the internet through the Adobe Flash based Xperiment software (http://www.xperiment.mobi).

In most previous studies^8^,^10^,^11^, two shapes were presented side-by-side together so that the participants could match the words (either presented visually or auditorily). Such a means of presentation allows the participant to compare the details of the shapes and notice any critical differences. However, such means of presentation would lead our participants to attend to the small difference between two patterns (e.g., an increased level of Amplitude) that they may not consider critical when viewing a single pattern^18^. Thus, on each trial in the current study, only a single visual pattern was presented on the monitor while two sounds were presented sequentially. This procedure also provides a more reserved measurement for fear of overestimating the reliability of the Bouba/Kiki effect^18^.

### Procedure

**Figure 1:**
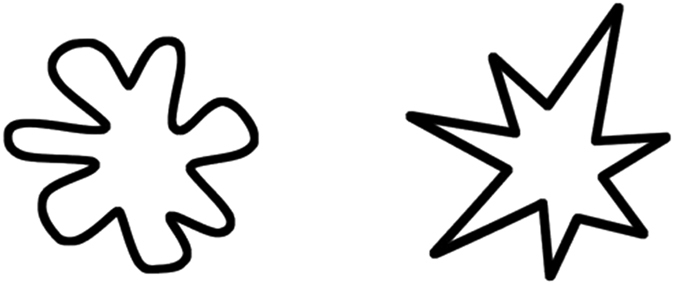
Two patterns used to demonstrate the Bouba/Kiki effect (e.g., Bremner *et al*.^10^). Most participants match the rounded one (left) to “Bouba” and the angular one (right) to “Kiki”.

Before starting the main experiment, the participants were requested to switch to full screen mode and confirmed that they could hear the sounds clearly (by typing in three digits that they heard^37^). In each trial, a RF pattern was presented in the center of the monitor, and participants had to judge whether “Bouba” or “Kiki” provided a better match for the pattern – both of them being presented auditorily and with the order counterbalanced on a trial-by-trial basis. Each participant had to complete 72 trials (6 Frequency × 4 Amplitude × 3 Spikiness) in a randomized order, as well as two original figures used in Bremner *et al*.’s study^10^ at the end (see Fig. 1).

## Results

In the first analysis, the agreement of participants’ matching judgments for each pattern in the US and Taiwanese group were assessed separately. That is, we used chi-square tests to determine whether participants in each group consistently judged a given pattern as better matching “Bouba” or “Kiki”, or not different from chance level (50%). For the two original patterns (Fig. 1), typical correspondences were observed between the Kiki/angular shape in both cultures (US group: 86.7%, *p* < 0.001; Taiwanese group: 90.8%, *p* < 0.001), and the Bouba/rounded shape only for Taiwanese (60.9%, *p* < 0.05) but not for the US group (only 50.6% of “Bouba” response, *p* = 0.87). The responses for each shape between two groups, however, were not significantly different (both *p*s ≥ 0.51).

For the RF patterns, a common trend was revealed in both the US and Taiwanese groups: Participants’ judgments shifted from “Bouba” to “Kiki” when each of the factors – Frequency, Amplitude, and Spikiness increased (see Fig. 4).

In order to examine whether all or only certain of the factors – Frequency, Amplitude, and Spikiness – significantly modulated participants’ responses, we used logistic regression in the lme4 (linear mixed effect) package^38^ (version 1.1-10) in R (version 3.2.1) to fit the data using maximum likelihood method to reach the optimal coefficient for each factor; and we applied parametric bootstrapping method 1,000,000 times in order to derive the standard error (SE) for each coefficient. Given the computed coefficient and SE for each factor, the 95% confidence interval (CI, the coefficient ±1.96 * SE) can be calculated. We fitted the data from the US and Taiwanese groups separately, and the CI for each factor can be compared^39^ (see Table 1). The results demonstrated that all three factors were significant predictors of participants’ performance in both the US and Taiwanese groups; however, differences between groups were observed. That is, when comparing the CIs of the coefficient between the two groups, the CIs of Frequency overlapped, but the CIs of the other two factors (i.e., Amplitude and Spikiness) did not. Specifically, the US group, had a higher coefficient for Amplitude but a lower coefficient for Spikiness as compared to the Taiwanese group.

In order to further confirm any cultural differences in the factors of Amplitude and Spikiness (but not Frequency), the goodness of fit of three logistic regression models were compared: Model 1 used four parameters (Frequency, Amplitude, Spikiness, and a constant) to fit the data combining the two groups; Model 2 used seven parameters (Frequency, Amplitude, Spikiness, ΔFrequency, ΔAmplitude, and ΔSpikiness, and a constant) to fit the data from the US and Taiwanese groups separately (Δ represents the difference of coefficients between the two groups); and finally, Model 3 used six parameters excluding ΔFrequency as compared to Model 2 (i.e., Frequency, Amplitude, Spikiness, ΔAmplitude, and ΔSpikiness, and a constant) to fit the data from the US and Taiwanese groups separately. In these models, only the ΔFrequency factor in Model 2 was not a significant predictor (*p* = 0.88; see Table 2), thus suggesting that the two groups had the same coefficient for the Frequency factor. When comparing the deviance values of each model in a pairwise manner (see Table 3), Model 1 had a significantly larger deviation than both Models 2 and 3, while the latter two models fit the participants’ performance equally well. This result once again suggests that different coefficients were required for the factors of Amplitude and Spikiness for the two groups of participants.

The age range of the North American participants was wider than the Taiwanese participants. We therefore compared the performance of the young North American (≤31 years old, N = 83) to all the Taiwanese participants (N = 88). The results of the models fitting remained; that is, the coefficients of Amplitude and Spikiness for the two groups of participants were different (see Supplementary Materials).

## Discussion

In the present study, RF patterns were systematically manipulated in order to test the crossmodal sound-shape correspondence between the words Bouba/Kiki on the one hand and rounded/angular shapes on the other. Three attributes of the RF patterns were manipulated – the frequency, amplitude, and spikiness of sinusoidal modulations along the circumference of a circle. The results demonstrated that the matching of both the North American and Taiwanese participants was modulated by all three factors; specifically, the participants were more likely to match an RF pattern to “Kiki” rather than “Bouba” when the frequency, amplitude, and spikiness increased. Here, we further demonstrated both cross-cultural commonalities and differences when matching RF patterns to Bouba or Kiki. That is, the responses of the North American and Taiwanese participants had similar weightings on the frequency factor. Nevertheless, the North American’s matching was weighted more heavily on the amplitude of the sinusoidal modulations than the Taiwanese, whereas the matching of the Taiwanese was weighted more heavily on spikiness of the sinusoidal modulations than the North Americans.

This is the first time that a robust cultural difference has been demonstrated in the Bouba/Kiki effect, which can be attributable to the different perceptual styles in Eastern and Western culture^29^. Specifically, Taiwanese participants, as an example of Eastern culture, are thought to process a visual pattern holistically. Therefore, they may attend to the overall contour composed by each lobe and the level of spikiness from each lobe could be summed together, thus giving rise to a stronger perception of spikiness that is associated with “Kiki”. Hence, the higher coefficient of spikiness in Easterners can be explained by their attending to overall contour. On the other hand, North Americans, as a Western culture, are suggested to process a visual pattern more analytically. That is, they are more likely to attend to the shape of individual lobes being continuous or distinctive from each other, in which the strength of the amplitude is the main factor to determine distinctiveness of each lobe. Hence, the higher coefficient of amplitude in Westerners can be explained by their attending to the shapes of individual lobes.

The cultural differences reported in the present study therefore suggest that experience of visual pattern perception is essential in the Bouba/Kiki effect. This result is consistent with a recent study testing people lacking of visual pattern vision: When mapping “Bouba” and “Kiki” to tactile stimuli with smooth or spiky shape (or texture), people with visual impairments (ranging from congenital blindness to partial sight) performed less reliably than did their sighted counterparts^40^. Combining these results therefore suggests that the Bouba/Kiki effect has a perceptual basis regarding pattern vision, which is consistent with the metaphorical perception account rather than the sound symbolism account reviewed in the Introduction.

In addition, our study is also the first to demonstrate that the participants’ matching shifted from “Bouba” to “Kiki” when the visual features of a pattern changed, step-by-step, along three dimensions. Conventionally, people are more likely to judge a pattern matching to “Kiki” rather than “Bouba” when its contour looks more angular, which is replicated in the present study by manipulating the factor of spikiness. Furthermore, we demonstrated two novel attributes that influenced the participants’ sound-shape matching as well. That is, the probability of matching a pattern to “Kiki” increases when its number of lobes increases (determined by the factor of frequency) and when each lobe becomes more distinctive (determined by the factor of amplitude). In turn, along each attribute, the dichotomous boundary to separate patterns that match to “Bouba” or “Kiki” can be revealed, and it would be possible to examine the perceptual mechanisms underpinning this sound-shape correspondences.

When increasing the frequency of RF patterns (a culture-general factor in the current study), for example, RF patterns started to be matched with “Kiki” when the frequency reached five. Interestingly, this is consistent with the boundary where RF patterns are processed by different pattern detection mechanisms. Specifically, previous research demonstrated that the visual system can pool the lobes of RF patterns efficiently into a global pattern representation up to the number of five; once the number of lobes increases further, each lobe is accessed independently and the information of the lobes is combined based on probability summation by the visual system^41^,^42^,^43^. In summary, low- and high-frequency RF patterns are processed by global and local pattern detection mechanisms, respectively^44^,^45^, and the dichotomous boundary is roughly located at the frequency of five. In the future, it will be possible to examine whether a visual pattern being processed globally versus locally can predict whether it is matched with “Bouba” or “Kiki”.

**Figure 2:**
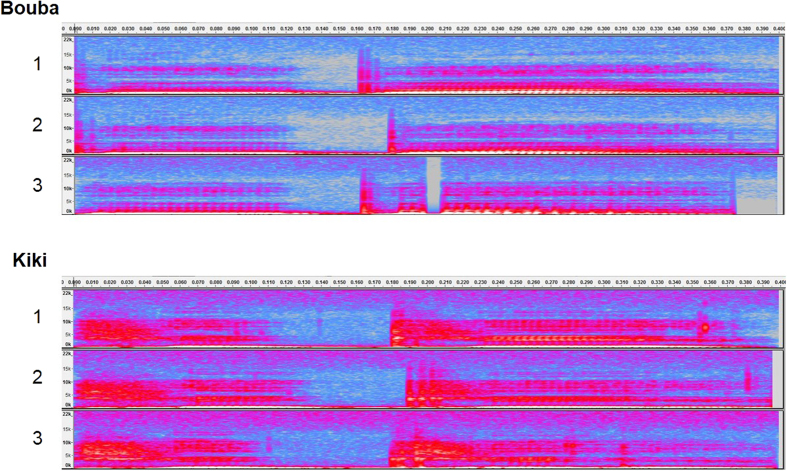
The spectrograms of the sound “Bouba” and “Kiki” (three examples for each) used in the present study. The graded colour from blue to red represents the higher energy distributed at particular sound frequencies (y-axis, ranging from 0 to 22,000 Hz) over time (x-axis, 0 to 400 ms). The spectrograms were plotted using Audacity (http://audacityteam.org/).

**Figure 3:**
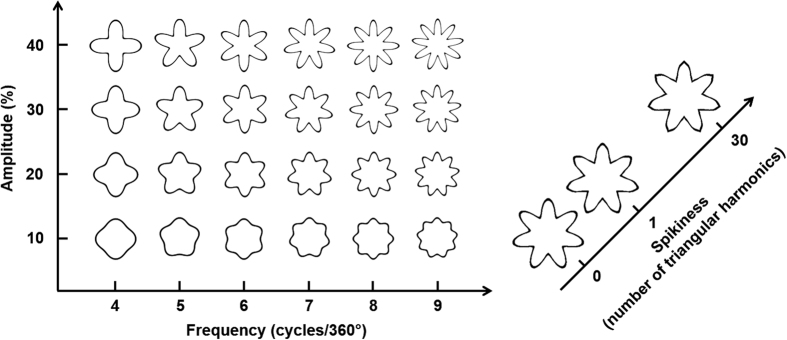
Radial frequency (RF) patterns used in this study. Three attributes were manipulated: Frequency, Amplitude, and the Spikiness of the sinusoidal modulations making-up the shape’s circumference.

**Figure 4:**
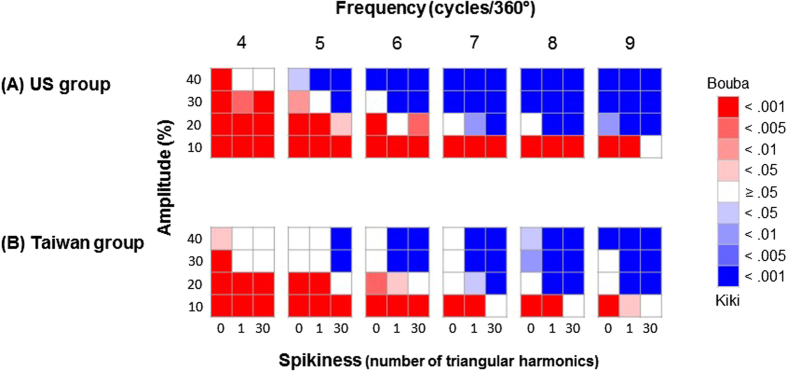
The results of (**A**) the US group and (**B**) Taiwanese group. Each cell represents its correspondence RF pattern. The RF patterns that were better matched with the sound “Bouba” are represented by red, while those that were better matched with the sound “Kiki” are represented by blue; finally, those that were undetermined are represented by white. The saturation of the colours represents the *p* values of the *Χ*^2^ tests.

How are the “Bouba” and “Kiki” sounds mapped on to global vs. local processing? In the spectrograms of these two types sounds (see Fig. 2), “Bouba” consisted of a shorter offset interval between the two syllables (mean: 43.7 ms) than “Kiki” (mean: 56.7 ms) after the length of the stimuli were equalized. The detailed analysis of acoustic features in the “Bouba” and “Kiki” effect, though, has been partly examined in previous studies^17^,^18^, requires future research.

The present study was conducted using an internet-based test, which constitutes a rapidly developing method nowadays^37^. The advantage of internet-based test mainly lies in that data from a large number of participants with various backgrounds (e.g., in different ages, races, countries, *etc.*) can be collected rapidly, therefore avoiding the potential critisism of homogeneity of participants (e.g., Western, Educated, Industrialised, Rich, and Democratic, WEIRD^46^). However, researchers may worry about the difficulty in controlling the parameters of stimulus presentation and the quality of the data collected. For the latter concern, as shown in the present study, the results from Taiwanese participants were generally consistent using three testing methods: group test, lab-based psychophysical test, and internet-based test (see Supplementary Experiments 1 and 2, and the main experiment), suggesting that internet-based test is a reliable method to a certain extent^37^,^47^. For the former concern, nevertheless, it is clear that the size of the visual stimuli and the loudness of the auditory stimuli were impossible to control precisely. Note that crossmodal correspondences refer to the phenomena whereby modality-specific features are matched relatively rather than absolutely, and the presentation of stimuli changing along the matching dimensions (e.g., higher- and lower-pitched tones) is necessary to demonstrate the crossmodal correspondence effects^1^. Given the fact that these two factors – visual size and auditory loudness – were held constant through the experiment for a given participant, they should be unlikely that they would have influenced the participants’ judgments systematically.

**Figure 5:**
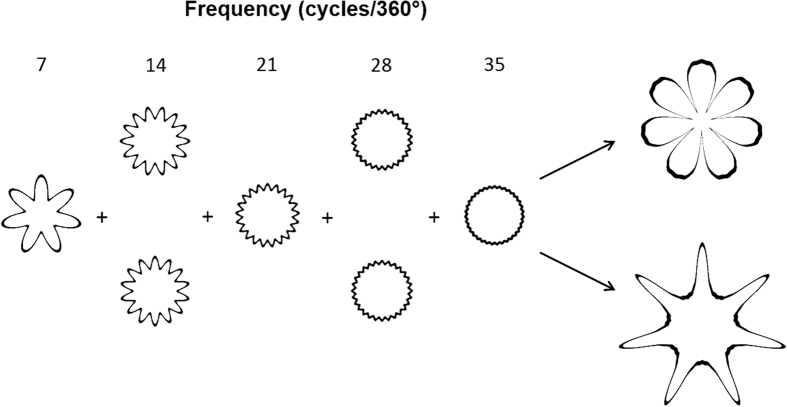
By integrating five RF patterns with the frequency (7, 14, 21, 28, and 35) and the corresponding amplitude (0.4, 0.2, 0.1, 0.05, and 0.025), two patterns that are similar to the original patterns in Fig. 1 can be created by changing the phase of the 2^nd^ and 4^th^ RF patterns. Specifically, in the rounded shape, the peak of the 1^st^, 3^rd^, and 5^th^ RF patterns and the trough of the 2^nd^ and 4^th^ RF patterns were aligned. The peaks of all five RF patterns were aligned for the angular shape.

Taken together, RF patterns are used for the first time here to demonstrate that the Bouba/Kiki effect reflects both cross-cultural commonality and differences, and the results suggest that this sound-shape correspondence is partly tuned by daily perceptual experience. In the present study, the visual stimuli were single RF patterns; nevertheless, future studies can utilize the fact that, by linearly combining several RF patterns, complex patterns that approach the original patterns used in the Bouba/Kiki effect can be created^48^ (see Fig. 5). The mechanism underlying the RF patterns suggests a mid-level crosstalk between visual processing (plausibly at V4^33^) and auditory processing^49^. Extending our understanding of crossmodal correspondences at different levels of processing may be helpful not only for understanding other cognitive functions (such as language acquisition^50^), but also for clinical application (such as in the development of sensory substitution devices for the blinds^51^).

## Additional Information

**How to cite this article**: Chen, Y.-C. *et al*. When “Bouba” equals “Kiki”: Cultural commonalities and cultural differences in sound-shape correspondences. *Sci. Rep.* **6**, 26681; doi: 10.1038/srep26681 (2016).

## Supplementary Material

Supplementary Information

## Figures and Tables

**Table 1:** The coefficient, SE, 95% confidence interval, *z* value, and *p* value (*μ* = 0) for each factor in the logistic regression analysis.

**Group**	**Factor**	**Coefficient**	**SE**	**95% confidence interval**		**z-values**	***p***
				**Upper**	**Lower**		
US	Frequency	0.394	0.014	0.421	0.366	28.21	<0.001
	Amplitude	0.089	0.002	0.093	0.085	39.88	<0.001
	Spikiness	0.014	0.002	0.017	0.011	8.76	<0.001
	Constant	−4.835	0.131	−4.579	−5.090	−37.04	<0.001
Taiwan	Frequency	0.373	0.018	0.407	0.339	21.26	<0.001
	Amplitude	0.066	0.003	0.071	0.061	24.32	<0.001
	Spikiness	0.024	0.002	0.028	0.020	11.61	<0.001
	Constant	−4.247	0.163	−3.928	−4.566	−26.11	<0.001

Note: The model of the logistic regression was glmer (Response ~1 + Frequency + Amplitude + Spikiness + (1|ID)).

**Table 2:** The coefficient, SE, *z* value, and *p* value (*μ* = 0) for each factor in the three logistic regression models.

**Model**	**Factor**	**Coefficient**	**SE**	**z-values**	***p***
1	Frequency	0.384	0.011	35.27	<0.001
	Amplitude	0.080	0.002	46.59	<0.001
	Spikiness	0.018	0.001	14.08	<0.001
	Constant	−4.597	0.102	−45.29	<0.001
2	Frequency	0.383	0.020	19.18	<0.001
	Amplitude	0.067	0.003	23.08	<0.001
	Spikiness	0.025	0.002	11.60	<0.001
	ΔFrequency	0.004	0.027	0.15	=0.88
	ΔAmplitude	0.021	0.004	5.44	<0.001
	ΔSpikiness	−0.010	0.003	−3.88	<0.001
	Constant	−4.563	0.180	−25.36	<0.001
3	Frequency	0.386	0.011	35.32	<0.001
	Amplitude	0.067	0.003	24.20	<0.001
	Spikiness	0.025	0.002	11.73	<0.001
	ΔAmplitude	0.021	0.004	5.82	<0.001
	ΔSpikiness	−0.011	0.003	−3.95	<0.001
	Constant	−4.567	0.168	−27.17	<0.001

Note: In *R*, Model 1 was glmer(Response ~1 + Frequency + Amplitude + Spikiness + (1|ID)); Model 2 was glmer(Response ~1 + Frequency + Amplitude + Spikiness + ΔFrequency + ΔAmplitude + ΔSpikiness + (1|ID) + (1|Nationality); Model 3 was glmer(Response ~1 + Frequency + Amplitude + Spikiness + ΔAmplitude + ΔSpikiness + (1|ID) + (1|Nationality).

**Table 3:** The comparison of the goodness fit of the three logistic regression models in Table 2.

**Model**	**df**	**Log Likelihood**	**Deviance**	**Model comparison**	***Χ***^**2**^ **test**	**df**	**p**
1	5	−9701.1	19402.2	1 vs. 2	54.5	4	<0.001
2	9	−9673.9	19347.7	2 vs. 3	0	1	=1
3	8	−9673.9	19347.7	1 vs. 3	54.5	3	<0.001
