# Supplementary material for: When “Bouba” equals “Kiki”: Cultural commonalities and cultural differences in sound-shape correspondences
Source: Sci Rep. 2016 May 27;6:26681. doi: 10.1038/srep26681 (PMC4882484; doi:10.1038/srep26681)
Supplement: Supplementary Information [file srep26681-s1.pdf]

SUPPLEMENTARY MATERIALS

When "Bouba" equals "Kiki": Cultural commonalities and cultural differences in sound-shape correspondences

Yi-Chuan Chen, Pi-Chun Huang, Andy Woods, and Charles Spence

Pilot Experiment 1

This is the first study to use radial frequency (RF) patterns[1] as visual stimuli when investigating sound-shape correspondences; specifically, the Bouba/Kiki effect[2-5]. We therefore designed a group test in order to verify whether the RF patterns chosen were, in fact, suitable to examine sound-shape correspondences.

Methods

Participants

Seventy-four undergraduates from the National Cheng Kung University in Taiwan took part in this study. They were naïve as to the purpose of the study and received additional course credits in return for their participation. The participants gave their informed consent prior to the experiment. All procedures were carried out according to the Declaration of Helsinki and were approved by the ethics committee of the psychology department in National Cheng Kung University.

Stimuli

Three attributes of the sinusoidal modulations along the circumference were manipulated (Figures S1A and S1B): There were five levels of Frequency (i.e., the number of sinusoidal modulations, 3, 5, 7, 11, or 15 cycle/circle), four levels of Amplitude of the sinusoidal modulations (0.1, 0.2, 0.3, or 0.4), and 3 levels of Spikiness (0, 1, or 30 triangular harmonics added to the sinusoidal modulations). Hence, a total of 60 RF patterns were created and used in this experiment. The RF patterns were presented using a black outline contour against a white background.

Procedure

All of the participants sat in the classroom and viewed the visual stimuli presented on the projector screen. Each participant was provided with a remote instant response system (IRS) response pad. They had to judge whether "Bouba" or "Kiki" (both presented auditorily for each visual stimulus) provided a better match for each RF pattern by pressing the corresponding button on their response pad. Each stimulus was presented until 95% of the participants responded. The participants' responses were recorded automatically by the system. It took about 20 minutes to complete the experiment.

Results and Discussion

The probability of participants' matching each RF pattern to "Bouba", "Kiki", or giving responses that were no different than chance level (50%) was examined using chi-square tests (see Figure S2). In summary, all of the three attributes modulated the sound-shape correspondence. When the Frequency increased from 3 to 11 cycles/circle, the associated sound shifted from "Bouba" to "Kiki". However, only for the intermediate Frequencies of 5 and 7 cycles/circle, did the associated sound shift from "Bouba" to "Kiki" when either the Amplitude or Spikiness was increased.

These results demonstrate that the manipulation of the frequency of the RF patterns had a significant main effect on the sound-shape correspondence: low-frequency patterns (e.g., 3) were associated with "Bouba", whereas high-frequency patterns (e.g., 11 and 15) were associated with "Kiki" instead. Interestingly, only for the RF patterns with an intermediate frequency (i.e., 5 and 7), did the amplitude and spikiness of the sinusoidal modulations influence the sound-shape correspondences. These results therefore suggest that frequency dominates over amplitude and spikiness in determining the observed sound-shape correspondences. Given these results, in the main experiment we therefore selected the RF patterns in the frequency range from 4 to 9.

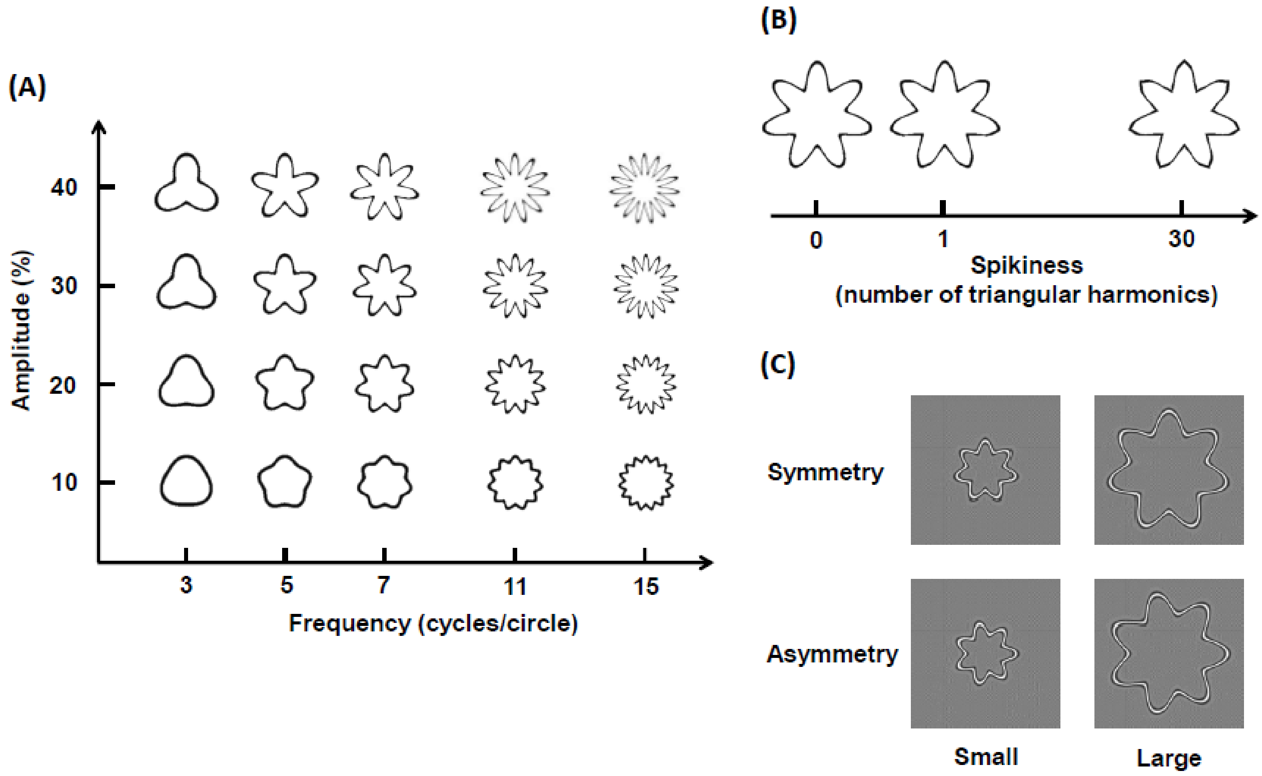

Figure S1. Radial frequency (RF) patterns used in the study. (A) RF patterns with increasing Frequency and Amplitude dimensions. (B) RF patterns with increasing Spikiness (i.e., increasing the number of triangular harmonics). (C) RF patterns at the two levels of Size (small or large) and Symmetry (left-right symmetry or asymmetry) in pilot Experiment 2.

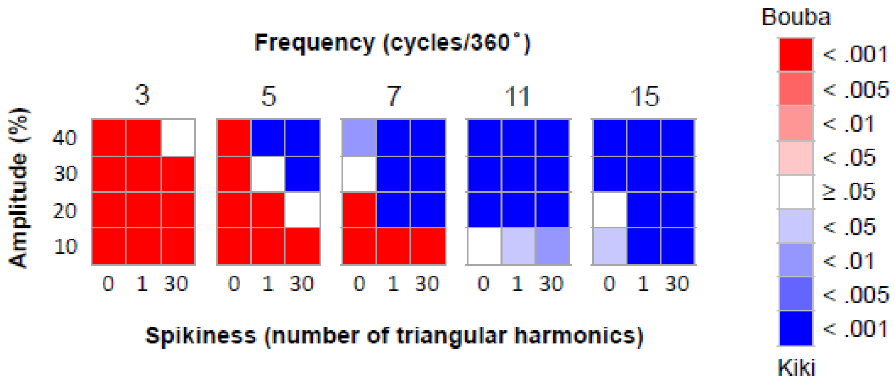

Figure S2. The results of pilot Experiment 1. Each cell represents its corresponding RF pattern. The RF patterns that were better matched with the sound “Bouba” are represented by red, while those that were better matched with the sound “Kiki” are represented by blue; finally, those that were undetermined are represented by white. The saturation of the colours represents the p values of the X^2 tests.

Pilot Experiment 2

The goal of pilot Experiment 2 was to verify that the three attributes, frequency, amplitude, and spikiness of the RF patterns were critical to the sound-shape correspondence in two regards: First, to provide a contrast, we manipulated two other attributes, the size and the symmetry of RF patterns, that may not influence participants' matching. Second, we wanted to replicate the results in a highly-controlled laboratory setting; that is, the fine-detailed features of the visual stimuli, and the amplitude of the auditory stimuli were exactly the same for each participant.

Methods

Participants

Ten participants (4 males, age range: 20-24 years) in National Cheng Kung University took part in this study. All had normal and corrected to normal vision by self-report. They were naïve as to the purpose of the study and were paid in return for their participation. The participants gave their informed consent before the experiment started. All of the procedures were carried out according to the Declaration of Helsinki and were approved by the National Cheng Kung University research ethics committee for human behavioral sciences.

Stimuli and procedure

The participants were seated in an experimental chamber at a viewing distance of 120 cm from the monitor. In this experiment, the cross-section luminance profile of the contour of RF patterns followed the fourth derivative of a Gaussian (used in the original study[1]; see Figure S1C). The equation is as follows:

$$\mathrm{D4(r)} = c\left[1 - 4\left(\frac{r - r_{mean}}{\sigma}\right)^2 + \frac{4}{3}\left(\frac{r - r_{mean}}{\sigma}\right)^2\right] \times e^{-\left(\frac{r-r_{mean}}{\sigma}\right)^2}$$

Where r_{mean} is the radius of the base circle, c is the contrast, σ determined the peak spatial frequency.

Five factors were manipulated: seven levels of Frequency (4, 5, 6, 7, 8, 9, and 10 cycles/circle), seven levels of Amplitude (0.10, 0.15, 0.20, 0.25, 0.30, 0.35, and 0.40), five levels of Spikiness (0, 1, 2, 5, and 30 numbers of triangular harmonics), two levels of Size (100 or 200 pixels of diameter, corresponding to 1.9° and 3.8° visual angles) and two levels of Symmetry (left-right symmetry or

asymmetry, Figure S1(C)). All of these RF patterns in these 980 conditions were presented once in each block in a randomized order. In each trial, a RF pattern was presented on the monitor, and the participants had to press the pre-designated key corresponding to "Bouba" or "Kiki". The corresponding keys were counterbalanced between participants. A sound rehearsal phase was presented every 10 trials in order to remind the participants the keys of "Bouba" and "Kiki" responses. It took about 40 minutes to finish a block, and each participant had to complete 10 blocks across several days.

Results and Discussion

The results of individual participants are shown in Figure S3. In general, the results demonstrated a consistent pattern as in pilot Experiment 1: The participants' matching of each RF pattern was shifted from "Bouba" to "Kiki" as the Frequency, Amplitude, and Spikiness of the sinusoidal modulations increased.

In order to verify whether all five of the manipulated factors significantly modulated participants' matching performance, logistic regression in the lme4 package[6] in R (version 3.2.1) was used to fit the data from the 10 participants (see Table S1). The results demonstrated that only the factors of Frequency, Amplitude, and Spikiness, but not Size and Symmetry, modulated the participants' matching of RF patterns to "Bouba" or "Kiki".

In summary, in two pilot experiments, we found that RF patterns are suitable to examine people's sound-shape correspondences. Specifically, participants matched a RF pattern to "Kiki" rather than "Bouba" when the frequency, amplitude, and spikiness of the sinusoidal modulations increased; by contrast, their performance was insensitive to the change of size and symmetry of the RF pattern, at least in the stimulus range manipulated here.

Taken together, among the three critical factors – frequency, amplitude, and spikiness, the latter two only effectively modulated the participants' responses under a certain range of frequencies (roughly 4-10). These pilot experiments are therefore critical when it comes to selecting the critical factors and the manipulated range of RF patterns to study sound-shape correspondences.

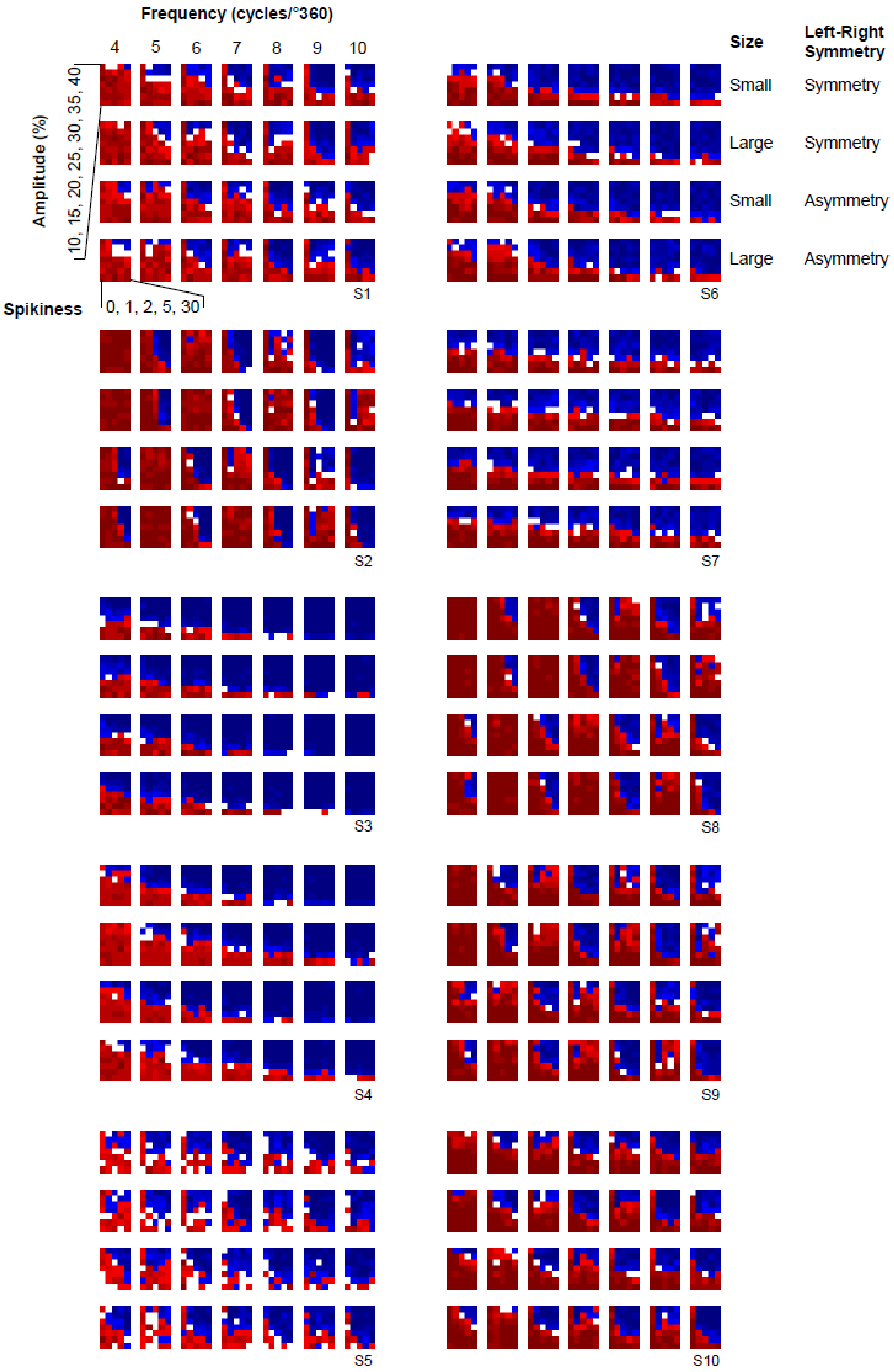

Figure S3. The results of 10 participants in pilot Experiment 2. Each cell represents its correspondence RF pattern. Red, blue, and white represents that the participants matched the RF patter to Bouba, Kiki, or equally in 10 trials, respectively. The darker colours represent higher probabilities.

Table S1. The coefficient, SE, *z* value and *p* value (μ = 0) for each factor in the logistic regression analysis.

Factor	Coefficient	SE	z-values	*p*
Frequency	0.343	0.132	2.60	< .01
Amplitude	12.308	1.090	11.29	< .001
Spikiness	0.026	0.007	3.67	< .001
Size	-0.003	0.003	-1.07	= 0.28
Symmetry	0.251	0.314	0.80	= 0.42
Constant	-5.514	0.526	-10.47	< .001

The model of the logistic regression was glmer(Response ~ 1 + Frequency + Amplitude + Spikiness + Size + Symmetry + (1 + Block + Frequency + Amplitude + Spikiness + Size + Symmetry|ID))

Young US vs Taiwanese participants

Given the fact that the participants in the North American group has a wider age range (19-68 years) than did the Taiwanese participants (18-22 years), we wanted to rule out the possibility that any cultural differences reported in the present study might be attributable to an age effect. The performance of young North Americans whose age was or younger than 31 years old (N = 83) was therefore compared to that of all Taiwanese participants (N = 88). The results were consistent with those reported in the main study – Model 1 had a significantly larger deviation than both Models 2 and 3, while the latter two models fit the participants' performance equally well (see Tables S2 & S3). These results suggest different coefficients of Amplitude and Spikiness factors were different for two groups, with only a common coefficient of Frequency factor was needed.

Table S2. The coefficient, SE, *z* value, and *p* value (μ = 0) for each factor in the three logistic regression models when comparing North American participants who were younger than 31 years old and all of Taiwanese participants.

Model	Factor	Coefficient	SE	z-values	*p*
1	Frequency	0.384	0.013	30.03	< .001
	Amplitude	0.077	0.002	38.45	< .001
	Spikiness	0.021	0.002	14.05	< .001
	Constant	-4.571	0.119	-38.49	< .001
2	Frequency	0.380	0.019	20.25	< .001
	Amplitude	0.067	0.003	23.76	< .001
	Spikiness	0.024	0.002	11.63	< .001
	ΔFrequency	0.012	0.029	0.41	= .68
	ΔAmplitude	0.023	0.004	5.20	< .001
	ΔSpikiness	-0.007	0.003	-2.17	< .05
	Constant	-4.598	0.229	-20.09	< .001
3	Frequency	0.386	0.013	30.07	< .001
	Amplitude	0.067	0.003	24.30	< .001
	Spikiness	0.025	0.002	11.71	< .001
	ΔAmplitude	0.022	0.004	5.31	< .001
	ΔSpikiness	-0.007	0.003	-2.25	< .05
	Constant	-4.594	0.199	-23.07	< .001

Note: In *R*, Model 1 was glmer(Response ~ 1 + Frequency + Amplitude + Spikiness + (1|ID)); Model 2 was glmer(Response ~ 1 + Frequency + Amplitude + Spikiness + ΔFrequency + ΔAmplitude + ΔSpikiness + (1|ID) + (1|Nationality); Model 3 was glmer(Response ~ 1 + Frequency + Amplitude + Spikiness + ΔAmplitude + ΔSpikiness + (1|ID) + (1|Nationality).

Table S3. The comparison of the goodness fit of the three logistic regression models in Table S2.

Model	df	Log Likelihood	Deviance	Model comparison	X^2 test	df	p
1	5	-7007.5	14015.0	1 vs. 2	35.7	4	< .001
2	9	-6989.6	13979.3	2 vs. 3	0.1	1	= .75
3	8	-6989.7	13979.4	1 vs. 3	35.6	3	< .001

REFERENCES

1. Wilkinson, F., Wilson, H. R., & Habak, C. Detection and recognition of radial frequency patterns. *Vision Res.* **38**, 3555-3568 (1998).
2. Bremner, A. J., et al. "Bouba" and "Kiki" in Namibia? A remote culture make similar shape-sound matches, but different shape-taste matches to Westerners. *Cognition.* **126**, 165-172 (2013).
3. Köhler, W. *Gestalt psychology*. New York, NY: Liveright (1929).
4. Köhler, W. *Gestalt psychology: An introduction to new concepts in modern psychology*. New York, NY: Liveright (1947).
5. Ramachandran, V. S., & Hubbard, E. M. Synaesthesia – a window into perception, thought and language. *J. Consciousness Stud.* **8**, 3-34 (2001).
6. Bates, D., et al. Linear Mixed-Effects Models using 'Eigen' and S4. (2015). Retrieved from https://cran.r-project.org/web/packages/lme4/lme4.pdf.
